# Lower right insular thickness is associated with more severe post-traumatic stress disorder symptoms among Ukrainian refugees

**DOI:** 10.3389/fpsyt.2026.1717949

**Published:** 2026-02-27

**Authors:** Julian Maciaszek, Anna Zimny, Przemysław Podgórski, Weronika Machaj, Julia Alejnikowa, Agnieszka Dybek, Marta Błoch, Błażej Misiak

**Affiliations:** 1 Department of Psychiatry, Wroclaw Medical University, Wroclaw, Poland; 2 Department of General and Interventional Radiology and Neuroradiology, Wroclaw Medical University, Wroclaw, Poland

**Keywords:** cortical volume, insula, migration, MRI, post-migration stress, PTSD, war trauma

## Abstract

**Introduction:**

Refugees often face traumatic experiences and ongoing post-migration stressors, increasing their risk for post-traumatic stress disorder (PTSD). However, no study to date has examined whether cortical thickness moderates the relationship between post-migration living difficulties (PMLDs) and PTSD symptoms. This study aimed to investigate if cortical thickness underlies vulnerability or resilience to PTSD in war-affected refugees.

**Methods:**

A total of 60 Ukrainian refugees (aged 27.8 ± 6.1 years, 81.7% females), who arrived in Poland after the 2022 Russian invasion, underwent assessment of behavioral and psychopathological characteristics together with MRI structural neuroimaging.

**Results:**

Refugees with PTSD had significantly reduced cortical thickness in the right insula compared to those without PTSD. They also reported higher levels of PMLDs and exposure to traumatic events. Logistic regression analyses revealed that decreased right insular cortex thickness and a greater number of traumatic experiences were associated with higher odds of PTSD symptoms after adjustment for age, sex, education, cigarette smoking status, and the current history of somatic diseases. A significant positive association of the interaction between the number of traumatic experiences and right insular cortex thickness with the odds of PTSD symptoms was also observed.

**Discussion:**

The findings imply that cortical thickness of the right insula might be associated with more severe PTSD symptoms among individuals exposed to traumatic experiences.

## 1 Introduction

Over 1% of the global population is currently forcibly displaced as a result of persecution, violence, or human rights abuses, and the number of refugees worldwide continues to grow (1). Refugees frequently experience traumatic experiences, leading to a high rate of mental disorders, including post-traumatic stress disorder (PTSD) (2, 3). In recent years, a growing focus has been placed on how the diverse and numerous stressors linked to adapting to a new country, language, and culture after migration affect mental health (4, 5). The term post-migration living difficulties (PMLDs) encompasses a range of factors, such as interpersonal challenges (e.g., discrimination, social isolation), emotional strain (e.g., loneliness), stress linked to the asylum process (e.g., lack of a work permit, visa uncertainty), and migration-related difficulties (e.g., separation from family, language difficulties) (5, 6). These PMLDs can significantly impact mental health; notably, the number of PMLDs has been shown to increase the likelihood of developing PTSD, regardless of prior traumatic experiences (7, 8).

Neuroimaging studies comparing trauma-exposed individuals with and without PTSD provide important insights into the neural mechanisms associated with both vulnerability to PTSD and resilience following trauma (9–11). These findings suggest that neurobiological alterations may follow distinct trajectories depending on whether PTSD develops. Within this framework, the insular cortex is increasingly conceptualized as a central hub integrating salience detection, interoceptive and autonomic signaling, and affective learning, thereby providing a mechanistic link to core PTSD symptom clusters (12). In one network study, PTSD has been framed as a disorder of aberrant salience processing, with the insula identified as a key node coordinating responses to biologically and emotionally relevant stimuli (12. In line with this view, one functional MRI study demonstrated exaggerated anterior insula activation to threat-related emotional faces in individuals with PTSD, consistent with heightened salience attribution and an intensified subjective sense of danger (13). Complementing these task-based findings, one resting-state study reported altered functional connectivity of insular subregions within the salience network, suggesting persistent abnormalities in intrinsic network organization related to hypervigilance (14). At the symptom level, one fear-learning study showed that greater avoidance severity in PTSD was associated with stronger insula engagement during extinction learning, implicating insula-mediated interoceptive representations in deficient safety learning (15). Finally, subsequent meta-analytic work reported reduced insular gray matter in PTSD (16). Identifying neurobiological correlates of psychopathology in traumatized refugees is essential, as it can help enable more effective prevention and treatment of psychiatric disorders in this population (17).

To date, several studies have examined the brain structure of traumatized refugees. One study reported reduced volumes in lateral prefrontal, parietal, and posterior midline regions in traumatized refugees: more pronounced in those with PTSD, compared to healthy controls (18). Another study found reduced cortical thickness in prefrontal and temporal regions in Vietnamese ex-political detainees relative to non-traumatized controls (19). A study involving North Korean refugees found that increased thickness in the right medial prefrontal cortex was significantly associated with lower levels of anxiety and somatization, but only in those who had experienced trauma without developing PTSD (20). Despite these findings, the characteristics of cortical thickness in refugees remain insufficiently understood. The studies have yielded inconclusive results, and one of them focused on gray matter volume, which does not strongly correlate with cortical thickness (21). Several structural imaging studies have been conducted on other traumatized populations, most of them comparing cortical thickness or gray matter volume between individuals with and without PTSD, with many indicating reductions particularly in frontotemporal regions (22–26).

To date, no study has examined the relationship between PMLDs and PTSD symptoms while accounting for the potential moderating role of cortical thickness (22–27). The present study aimed to determine whether cortical thickness is associated with vulnerability and resilience to PTSD symptoms following war-related migration. An additional aim was to examine whether cortical thickness moderates the association of traumatic events and PMLDs with the severity of PTSD symptoms in refugees. We hypothesized that refugees with PTSD symptoms differ from those without PTSD symptoms in terms of cortical thickness and that cortical thickness moderates associations of traumatic experiences and PMLDs with PTSD symptoms among refugees.

## 2 Materials and methods

Participants were 60 refugees from Ukraine who crossed the Polish-Ukrainian border after the Russian invasion on 24 February 2022 (11 males and 49 females, aged 27.8 ± 6.1 years). Recruitment was conducted via social media and institutional websites. The study protocol was approved by the Bioethics Committee at Wroclaw Medical University (approval number: KB-97/2023), and all participants gave written informed consent. Exclusion criteria were: (1) age <18 or >65; (2) neurological conditions; (3) cognitive impairment; (4) substance/alcohol dependence (excluding nicotine); and (5) ICD-10 diagnoses including organic mental disorders (F00–F09), schizophrenia-spectrum disorders (F20–F29), bipolar I disorder (F31), and severe personality disorders (F60–F69).

The Impact of Event Scale-Revised (IES-R) was used to identify PTSD-related symptoms (28–31). Scores ≥33 were used as the threshold for probable PTSD (27). The Post-Migration Living Difficulties Checklist (PMLDC) assessed 17 difficulties over the past 12 months (33–36. The Traumatic Experiences Checklist (TEC) measured lifetime exposure to 29 types of traumatic experiences (29, 32, 33). The Cronbach’s alpha for the IES-R total score was 0.93 in the present study.

The GAD-7 was used to assess the severity of generalized anxiety symptoms (34). This self-report measure comprises seven items rated on a 4-point scale from 0 (“not at all”) to 3 (“nearly every day”), yielding a total score ranging from 0 to 21, with higher scores reflecting greater anxiety symptom severity. In the present study, the GAD-7 demonstrated good internal consistency, with a Cronbach’s alpha of 0.87.

Depressive symptom severity was assessed using the Beck Depression Inventory (BDI), a 21-item self-report questionnaire measuring characteristic cognitive, affective, and somatic symptoms of depression (35). Each item is scored from 0 to 3, where higher values indicate more severe depressive symptomatology. Total BDI scores range from 0 to 63, with higher scores reflecting greater depressive symptom severity. The internal consistency of the BDI in the current sample was high (Cronbach’s alpha = 0.89).

The severity of insomnia symptoms was evaluated using the Insomnia Severity Index (ISI) (36). The ISI consists of seven items rated on a 5-point scale from 0 (“not at all”) to 4 (“very severe”), resulting in a total score ranging from 0 to 28, with higher scores indicating more severe insomnia symptoms. In the present study, the ISI demonstrated excellent internal consistency (Cronbach’s alpha = 0.90).

Anatomical imaging was acquired on a 3T Philips Ingenia scanner with a high-resolution sagittal T1-weighted sequence. Data processing focused on cortical thickness using the Desikan–Killiany atlas (37) implemented in CAT12/SPM12. Preprocessing included SANLM denoising, bias correction, affine registration, and unified segmentation (38, 39), skull stripping, DK parcellation (37), local intensity transformation, AMAP segmentation, partial volume estimation (40), DARTEL normalization, and surface-based processing to the FreeSurfer template; thickness was computed for 68 cortical regions (37). Quality assurance included visual inspection and CAT12 automatic QC (IQR > 84%).

### 2.1 Statistics

Spearman correlations assessed associations among continuous variables. Group differences used t tests or Mann–Whitney U tests for continuous variables and χ² tests for categorical variables. Shapiro–Wilk tested normality. Multiple comparisons across cortical regions were controlled with Benjamini–Hochberg FDR at 25%. Logistic regression tested associations of PTSD status with number of traumatic events, PMLDs, and cortical thickness in regions with significant group differences. Model 1 included main effects; Model 2 added interactions (PMLDs×thickness; traumatic events×thickness); Model 3 added sociodemographic covariates (age, sex, education years, cigarette smoking, somatic disease) and Model 4 added clinical covariates (generalized anxiety symptoms, depressive symptoms, insomnia). Predictors were z-standardized; multicollinearity was checked via VIF (<4) (41). Analyses were performed in JASP (2025). Visualization of the interaction between traumatic exposure and right insular cortex thickness was performed in R, using model-based predicted probabilities and 95% confidence intervals derived from the fitted logistic regression model.

## 3 Results

**Figure 1:**
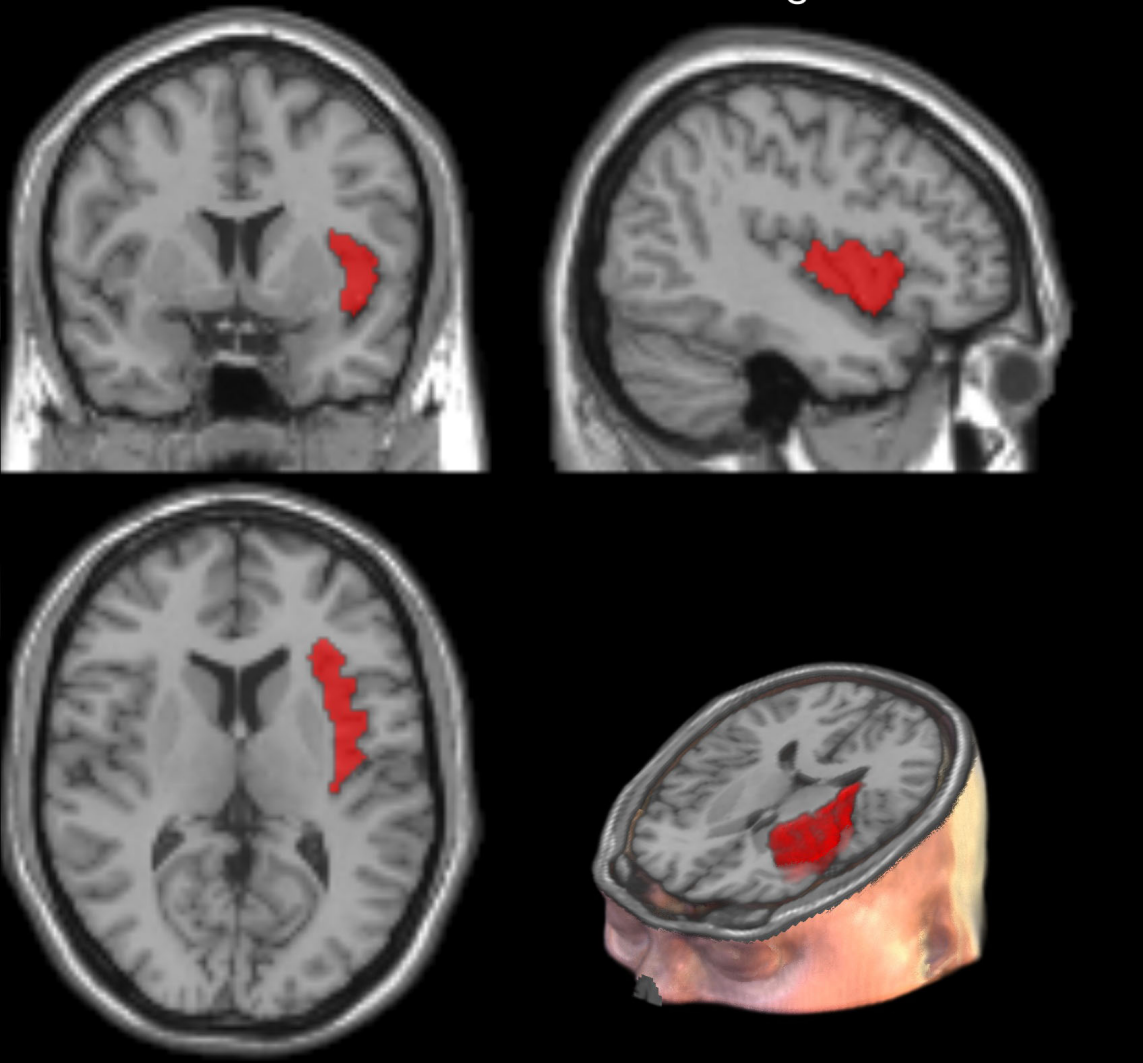
Cortical thickness in the right insular cortex.

**Figure 2:**
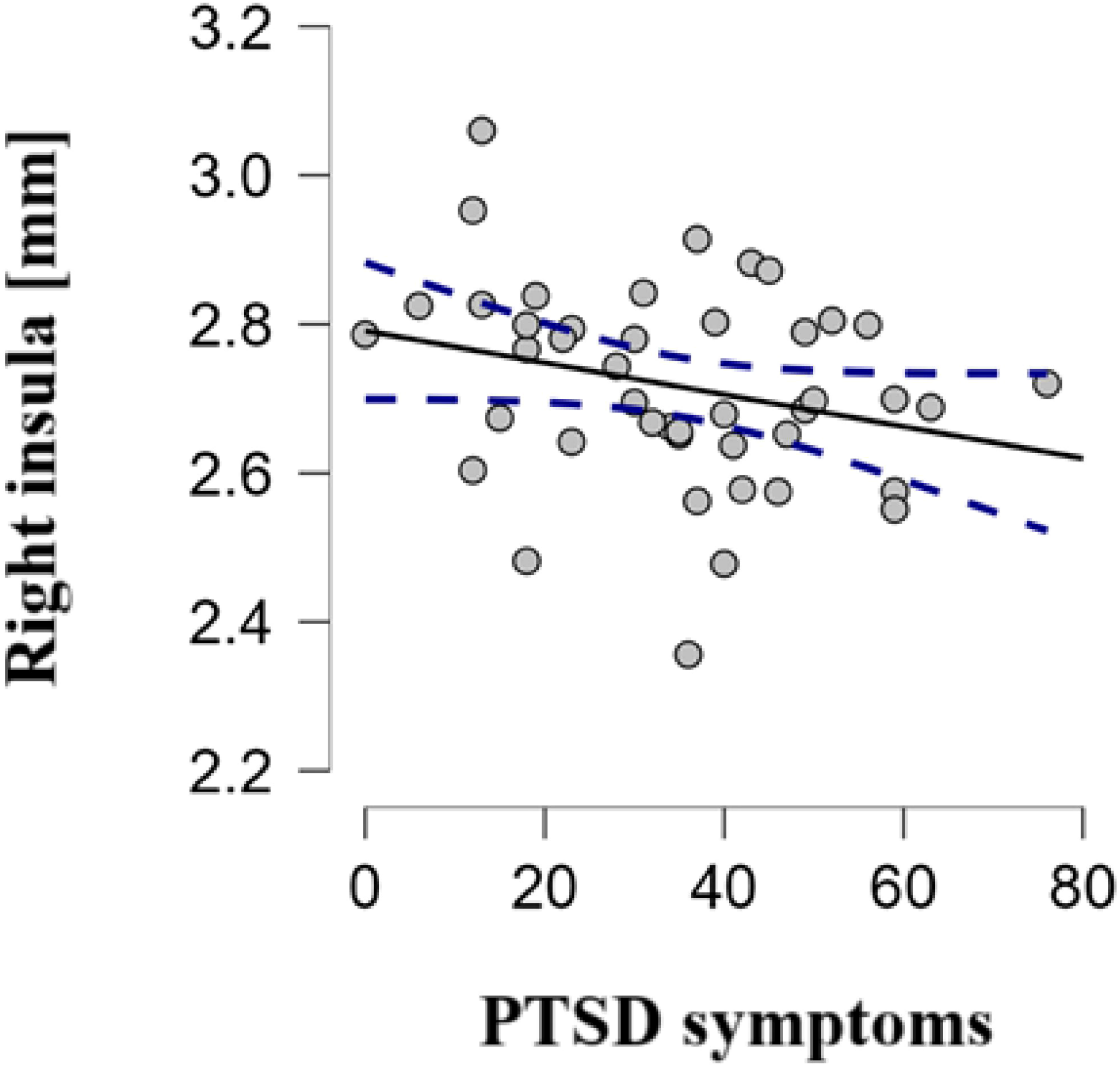
Correlation between right insular cortex thickness and PTSD symptoms in all participants.

**Figure 3:**
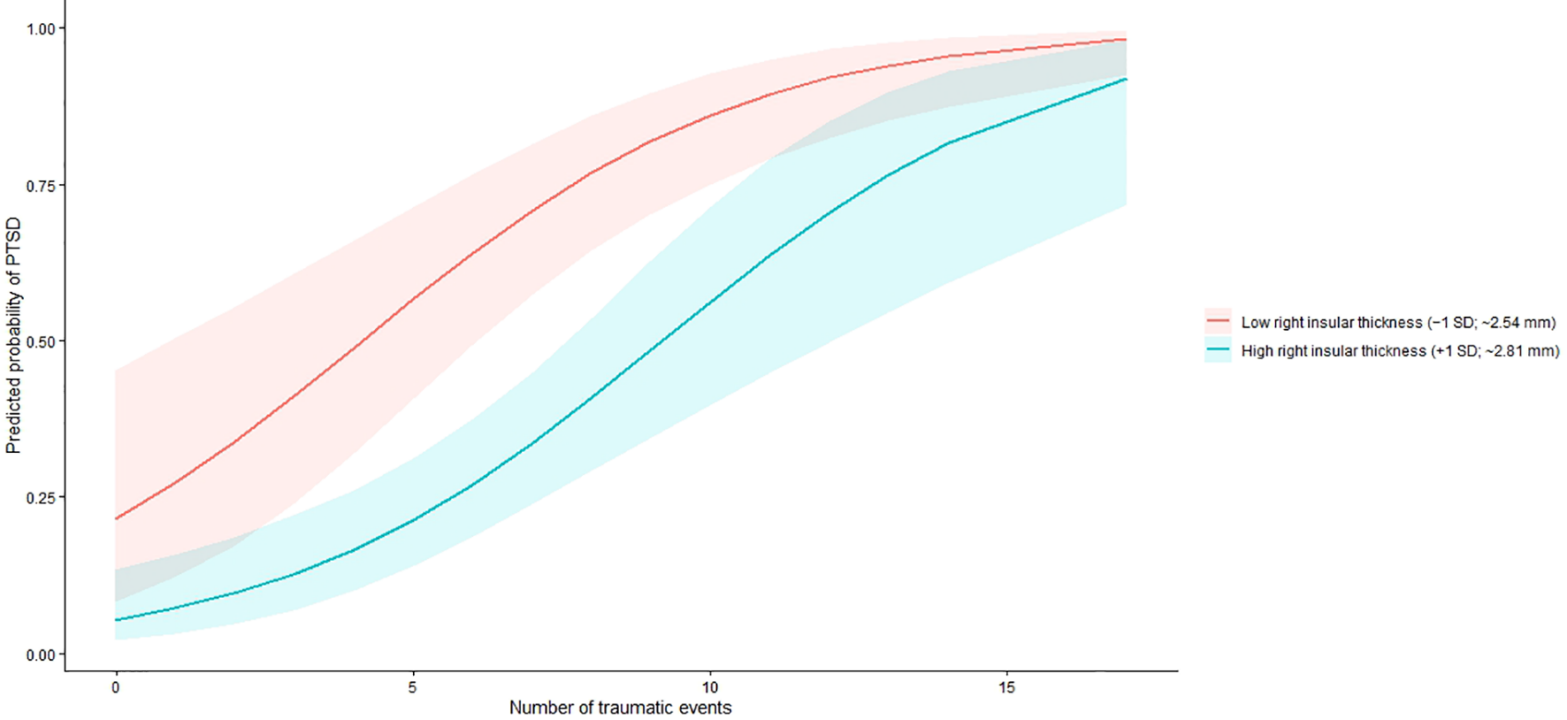
Moderating effect of right insular cortex thickness on the association between traumatic exposure and post-traumatic stress disorder (PTSD). Predicted probabilities of PTSD are plotted against the number of traumatic events for individuals with low (−1 SD) and high (+1 SD) right insular cortex thickness. Lower right insular thickness was associated with greater vulnerability to PTSD, as reflected by a steeper increase in PTSD probability with increasing traumatic exposure among individuals with reduced insular thickness. Shaded areas represent 95% confidence intervals. Predicted values were derived from a logistic regression model adjusted for age, sex, years of education, cigarette smoking status, presence of somatic diseases, generalized anxiety symptoms, depressive symptoms, and insomnia severity.

The analyses included 60 participants (aged 27.9 ± 6.2 years, 85.0% females). **Table 1** shows the general characteristics of refugees. Higher total IES-R scores and subscale scores were significantly correlated with higher PMLDC and TEC scores. Individuals with PTSD symptoms did not differ from those without PTSD symptoms in age, sex, education, somatic disease presence, or smoking status. However, those with PTSD symptoms had significantly reduced cortical thickness in the right insula after multiple-comparison correction (2.5 ± 0.1 mm vs. 2.8 ± 0.1 mm, p = 0.012). **Figure 1** presents visualization of right insular thickness generated using Desikan–Killiany atlas ROIS overlayed on a volumetric 3D T1-weighted MR images. They also had significantly higher GAD-7 (11.4 ± 4.3 vs 5.3 ± 4.1), BDI (16.4 ± 8.5 vs 10.7 ± 5.4), PMLDC (24.1 ± 9.0 vs. 17.5 ± 8.8) and TEC scores (8.4 ± 4.4 vs. 5.6 ± 3.2). The bivariate correlations between PMLDC, traumatic events and symptoms of PTSD are shown in **Table 2**. **Figure 2** shows correlation between right insular cortex and between severity of PTSD symptoms. The right insular cortex thickness correlated inversely with total IES-R (r = −0.245, p = 0.025). The results of logistic regression analysis are presented in **Table 3**. In logistic regression (adjusted model), decreased right insular thickness and a greater number of traumatic experiences were associated with higher odds of a positive IES-R screen; the traumatic experiences × right insular thickness interaction was also significant (odds ratio < 1.0). **Figure 3** shows the predicted probability of PTSD as a function of traumatic exposure at low (−1 SD) and high (+1 SD) levels of right insular cortex thickness, indicating a significant interaction between traumatic exposure and right insular thickness.

**Table 1:** The general characteristics of refugees.

Variable	Total sample (n = 60)	Refugees with PTSD (n=34)	Refugees without PTSD (n=22)	p
Age, years	27.9 ± 6.2	29.0 ± 5.5	29.0 ± 7.1	0.582
Sex, female	51 (81.7%)	27 (84.4%)	21 (80.8%)	0.667
Education, years	17.2 ± 2.2	13.5 ± 3.6	14.2 ± 2.5	0.756
Cigarette smoking, yes	15 (25.0%)	8 (25%)	6 (23.1%)	0.832
Somatic disease, yes	23 (38.3%)	17 (53.1%)	6 (23.1%)	0.092
PMLDC, post-migration living difficulties checklist	22.2 ± 8.4	8.4 ± 4.4	5.6 ± 3.2	**0.002**
TEC, The Traumatic Experience Checklist (number of events)	7.2 ± 4.1	24.1 ± 9.0	17.5 ± 6.8	**<0.001**
IES-R, total score	34.9 ± 16.8	46.8 ± 10.6	18.9 ± 9.3	**<0.001**
IES-R, positive scoring	34 (56.7%)	34.0(100%)	0.0 (0%)	**<0.001**
IES-R, intrusion	12.8 ± 6.9	17.7 ± 5.2	7.0 ± 4.3	**<0.001**
IES-R, avoidance	10.1 ± 5.2	15.8 ± 4.4	6.1 ± 3.2	**<0.001**
IES-R, hyperarousal	11.6 ± 5.8	13.2 ± 4.1	5.8 ± 4.2	**<0.001**
BDI	13.3 ± 7.6	16.4 ± 8.5	10.7 ± 5.4	**<0,001**
GAD-7	8.5 ± 5.0	11.4 ± 4.3	5.3 ± 4.1	**0.029**
ISI	19.0 ± 7.8	19.4 ± 4	17.5	**0.626**

Data expressed as mean ± SD or n (%).

BDI, Beck Depression Inventory; GAD-7, General Anxiety Disorder-7; IES-R, Impact of Events Scale – Revised; ISI, Insomnia Severity Index; PMLDC, Post Migration Living Difficulties Checklist; TEC, The Traumatic Experience Checklist.

Significant p-values after Benjamini–Hochberg correction were marked with bold characters (p ≤ 0.029).

**Table 2:** The bivariate correlations between post-migration living difficulties, traumatic events and symptoms of post-traumatic stress disorder.

Variable	PMLDC	TEC
IES-R, total score	r = 0.443, **p = 0.002**	r = 0.532, **p < 0.001**
IES-R, positive scoring	r = 0.295, **p = 0.003**	r = 0.332, **p = 0.042**
IES-R, intrusion	r = 0.411, **p = 0.007**	r = 0.470, **p = 0.001**
IES-R, avoidance	r = 0.421, **p = 0.006**	r = 0.329, **p = 0.024**
IES-R, hyperarousal	r = 0.423, **p = 0.004**	r = 0.525, **p < 0.001**

Significant correlations (p < 0.05) are marked in bold.

IES-R, Impact of Events Scale – Revised; PMLDC, Post Migration Living Difficulties Checklist; TEC, The Traumatic Experience Checklist.

**Table 3:** The results of logistic regression analysis testing for differential associations of the presence of post-traumatic stress disorder symptoms with post-migration living difficulties, a history of traumatic experiences, and cortical thickness.

Model (Nagelkerke R^2^)	Independent variable	O.R.	p	VIF	95% CI
Model 1 (0.343)	Post-migration living difficulties	1.030	0.056	1.005	-0.002 - 0.159
	Traumatic events	3.532	0.053	1.753	-0.142 – 0.463
	Right insular cortex	0.502	0.055	1.006	-13.853 – 1.753
Model 2 (0.432)	Post-migration living difficulties	1.026	0.123	1.003	-2.532 – 30.311
	Traumatic events	3.632	0.062	1.853	-0.153 – 0.453
	Right insular cortex	-0.553	0.072	1.041	-13.532 – 1.253
	Post-migration living difficulties × Right insular cortex	2.252	0.153	1.053	-0.253 – 3.532
	Traumatic events × Right insular cortex	0.542	0.051	1.885	-13.532 – 8.532
Model 3 (0.449)	Post-migration living difficulties	1.031	0.232	1.532	-0.053 – 0.178
	Traumatic events	3.643	**0.045**	1.642	-0.164 – 0.464
	Right insular cortex	-0.453	**0.042**	1.152	-13.642 – -0.264
	Post-migration living difficulties × Right insular cortex	1.164	0.132	1.064	-0.264 – 2.065
	Traumatic events × Right insular cortex	0.685	**0.044**	1.953	-16.643 – 6.643
	Age	1.064	0.664	1.153	-0.154 – 0.164
	Sex	0.663	0.553	1.252	-2.533 – 1.364
	Education years	0.956	0.253	1.153	-0.453 – 0.184
	Cigarette smoking status	0.853	0.563	1.153	-1.133 – 2.493
	Somatic disease	1.463	0.363	1.063	-0.753 – 2.632
Model 4 (0.572)	Post-migration living difficulties	-0.033	0.862	1.642	0.667 – 1.40.4
	Traumatic events	2.223	**0.034**	1.346	0.002 – 1.584
	Right insular cortex	-3.387	**0.047**	1.643	0.001 – 4.957
	Post-migration living difficulties × Right insular cortex	4.547	0.120	1.256	0.307 – 2.898
	Traumatic events × Right insular cortex	0.721	**0.036**	2.543	0.021 – 1.113
	Age	-0.017	0.897	1.536	0.763 – 1.267
	Sex	0.684	0.697	1.959	0.063 – 6.191
	Education years	-0.518	0.183	1.736	0.278 – 1.277
	Cigarette smoking status	-3.562	0.151	1.01	0.008 – 3.687
	Somatic disease	0.555	0.716	1.281	0.087 – 3.693
	Generalized anxiety symptoms	1.210	0.027	1.952	1.146 – 9.825
	Depressive symptoms	-0.048	0.856	2.302	0.565 – 1.607
	Insomnia	-0.049	0.688	1.992	0.751 – 1.208

Significant effects (p < 0.05) are marked in bold.

## 4 Discussion

We found that lower right insular thickness and a greater number of traumatic events were associated with more severe PTSD symptoms among Ukrainian refugees. A significant interaction between traumatic experiences and right insular thickness suggests that greater right insular thickness may protect against the impact of trauma on PTSD symptoms. These findings align with prior work reporting reduced insular thickness/volume in PTSD across other populations (19, 42–44) and with evidence linking higher PTSD severity and greater combat exposure to reduced insular thickness (45). The insula plays a key role in interoception, emotional awareness, and homeostatic regulation as part of the salience network (45–47). The specificity of the observed effect to the right insula is biologically plausible and consistent with prior neuroimaging literature indicating functional and structural lateralization of insular processes relevant to PTSD. The right insula has been preferentially implicated in interoceptive awareness and autonomic arousal, supporting the rapid detection and integration of salient and threatening stimuli (48, 49). Consistent with our findings, structural neuroimaging studies have repeatedly shown reduced insular gray matter in PTSD, as summarized in a meta-analysis of voxel-based morphometry studies (50). Extending this structural perspective, network-based models conceptualize PTSD as a disorder of disrupted salience processing, identifying the insula as a central hub integrating biologically and emotionally relevant information (51). Functional MRI findings further converge with our results by showing exaggerated anterior insula reactivity to threat-related cues in PTSD (52). At the network level, altered intrinsic connectivity of insular subregions within the salience network has been linked to persistent hypervigilance (53), a core PTSD feature. Additionally, evidence from fear extinction paradigms implicating insula engagement in avoidance symptom severity (54) is consistent with our finding that reduced right insular thickness was associated with greater PTSD symptom vulnerability. Meta-analytic and task-based studies show insular hyperactivation and altered connectivity in PTSD across paradigms (55–58). Therapy-related changes in insula–amygdala connectivity have also been reported (59, 60). In another study it was found that gray matter volume within the dorsal anterior cingulate and dorsomedial prefrontal cortex was linked to posttraumatic stress symptoms via dispositional optimism, supporting a vulnerability-based model of PTSD (61). This finding implicates salience-related cortical networks in individual susceptibility to PTSD. Extending this framework, reduced right insular cortical thickness observed in our study may represent a complementary structural vulnerability within the same network. In line with this interpretation, a recent review showed that PTSD is characterized by increased insular activation and disrupted insula-centered salience network connectivity compared with major depressive disorder and healthy controls (62).

This study has several limitations. The sample size was relatively small and predominantly female, which may limit generalizability, particularly to male refugees. The migrant group was heterogeneous with respect to war-related experiences, and reasons for migration were not assessed, potentially introducing variability. Self-reported symptom measures were used rather than structured clinical interviews. A further limitation is that cortical thickness was examined using region-based estimates derived from the Desikan–Killiany atlas rather than vertex-wise or data-driven morphometric analyses, which may have reduced sensitivity to detect more spatially localized cortical effects. A limitation of this study is the use of a relatively liberal false discovery rate threshold .2(q = 0.25), reflecting the exploratory nature of the interaction-based neuroimaging analyses, which increases the risk of false-positive findings. However, this approach is consistent with prior psychiatric neuroimaging literature indicating that interaction effects are typically small and underpowered, and that overly conservative correction strategies may increase the risk of Type II error (63). We focused primarily on structural brain changes without assessing functional correlates. The cross-sectional design prevents causal inferences. Future research should employ longitudinal and multimodal designs integrating structural and functional imaging with clinical assessment to better understand the neurobiological mechanisms of PTSD among refugees (64–68).

In summary, right insular cortical thickness may be associated with more severe PTSD symptoms among refugees. Reduced right insular thickness may be associated with greater PTSD risk following trauma exposure, underscoring the importance of considering brain structure in risk stratification and intervention. Future studies should examine whether right insular thickness prospectively predicts PTSD trajectories following trauma exposure, using longitudinal designs that can clarify temporal and potentially causal relationships. In addition, integrating vertex-wise morphometry with functional and connectivity measures of the salience network may help determine whether preserved right insular structure confers resilience by supporting more adaptive interoceptive and threat-processing responses in traumatized refugee populations.

## Data Availability

The raw data supporting the conclusions of this article will be made available by the authors, without undue reservation.
