# Supplementary material for: Lower right insular thickness is associated with more severe post-traumatic stress disorder symptoms among Ukrainian refugees
Source: Front Psychiatry. 2026 Feb 27;17:1717949. doi: 10.3389/fpsyt.2026.1717949 (PMC12982366; doi:10.3389/fpsyt.2026.1717949)
Supplement: Supplementary file 1 [file Table1.docx]

**Supplementary Table 1.**  The comparison of general characteristics and cortical thickness depending on the presence of post-traumatic stress disorder symptoms.

| Variable | Refugees with PTSD (n=34) | Refugees without PTSD (n=22) | *p* |  |
| --- | --- | --- | --- | --- |
| Left caudal anterior cingulate cortex, mm | 2.3 ± 0.2 | 2.3 ± 0.2 | 0.164 |  |
| Right caudal anterior cingulate cortex, mm | 2.3 ± 0.2 | 2.3 ± 0.2 | 0.366 |  |
| Left caudal middle frontal gyrus, mm | 2.6 ± 0.1 | 2.7 ± 0.1 | 0.294 |  |
| Right caudal middle frontal gyrus, mm | 2.7 ± 0.1 | 2.7 ± 0.1 | 0.928 |  |
| Left cuneus, mm | 2.0 ± 0.1 | 2.0 ± 0.1 | 0.273 |  |
| Right cuneus, mm | 2.0 ± 0.1 | 2.0 ± 0.1 | 0.772 |  |
| Left entorhinal cortex, mm | 2.8 ± 0.2 | 2.7 ± 0.3 | 0.430 |  |
| Right entorhinal cortex, mm | 2.7 ± 0.2 | 2.6 ± 0.3 | 0.209 |  |
| Left fusiform gyrus, mm | 2.5 ± 0.1 | 2.5 ± 0.1 | 0.392 |  |
| Right fusiform gyrus, mm | 2.5 ± 0.1 | 2.5 ± 0.1 | 0.266 |  |
| Left inferior parietal lobe, mm | 2.4 ± 0.1 | 2.4 ± 0.1 | 0.622 |  |
| Right inferior parietal lobe, mm | 2.4 ± 0.1 | 2.4 ± 0.1 | 0.725 |  |
| Left inferior temporal gyrus, mm | 2.4 ± 0.1 | 2.5 ± 0.1 | 0.323 |  |
| Right inferior temporal gyrus, mm | 2.5 ± 0.1 | 2.5 ± 0.1 | 0.467 |  |
| Left isthmus cingulate gyrus, mm | 1.9 ± 0.1 | 1.9 ± 0.1 | 0.441 |  |
| Right isthmus cingulate gyrus, mm | 1.9 ± 0.1 | 2.0 ± 0.1 | 0.249 |  |
| Left lateral occipital lobe, mm | 2.2 ± 0.1 | 2.2 ± 0.1 | 0.451 |  |
| Right lateral occipital lobe, mm | 2.2 ± 0.1 | 2.2 ± 0.1 | 0.796 |  |
| Left lateral orbitofrontal cortex | 2.5 ± 0.1 | 2.5 ± 0.1 | 0.365 |  |
| Right lateral orbitofrontal cortex, mm | 2.5 ± 0.1 | 2.4 ± 0.1 | 0.055 |  |
| Left lingual gyrus, mm | 2.1 ± 0.1 | 2.1 ± 0.1 | 0.945 |  |
| Right lingual gyrus, mm | 2.1 ± 0.1 | 2.1 ± 0.1 | 0.846 |  |
| Left medial orbitofrontal cortex, mm | 2.1 ± 0.1 | 2.1 ± 0.1 | 0.555 |  |
| Right medial orbitofrontal cortex, mm | 2.2 ± 0.1 | 2.1 ± 0.1 | 0.852 |  |
| Left middle temporal gyrus, mm | 2.7 ± 0.1 | 2.7 ± 0.1 | 0.554 |  |
| Right middle temporal gyrus, mm | 2.7 ± 0.1 | 2.7 ± 0.1 | 0.884 |  |
| Left parahippocampal gyrus, mm | 2.4 ± 0.1 | 2.4 ± 0.1 | 0.080 |  |
| Right parahippocampal gyrus, mm | 2.4 ± 0.1 | 2.4 ± 0.1 | 0.925 |  |
| Left paracentral lobule, mm | 2.5 ± 0.1 | 2.5 ± 0.1 | 0.853 |  |
| Right paracentral lobule, mm | 2.4 ± 0.1 | 2.4 ± 0.1 | 0.705 |  |
| Left pars opercularis, mm | 2.6 ± 0.1 | 2.6 ± 0.1 | 0.602 |  |
| Right pars opercularis, mm | 2.6 ± 0.1 | 2.6 ± 0.1 | 0.374 |  |
| Left pars orbitalis, mm | 2.5 ± 0.1 | 2.5 ± 0.2 | 0.982 |  |
| Right pars orbitalis, mm | 2.5 ± 0.1 | 2.5 ± 0.1 | 0.922 |  |
| Left pars triangularis, mm | 2.5 ± 0.1 | 2.5 ± 0.1 | 0.523 |  |
| Right pars triangularis, mm | 2.5 ± 0.1 | 2.5 ± 0.1 | 0.514 |  |
| Left pericalcarine cortex, mm | 2.0 ± 0.1 | 1.9 ± 0.1 | 0.373 |  |
| Right pericalcarine cortex, mm | 2.0 ± 0.1 | 2.0 ± 0.1 | 0.383 |  |
| Left postcentral gyrus, mm | 2.2 ± 0.1 | 2.2 ± 0.1 | 0.742 |  |
| Right postcentral gyrus, mm | 2.2 ± 0.1 | 2.2 ± 0.1 | 0.726 |  |
| Left posterior cingulate cortex, mm | 2.2 ± 0.1 | 2.2 ± 0.1 | 0.816 |  |
| Right posterior cingulate cortex, mm | 2.2 ± 0.1 | 2.2 ± 0.1 | 0.454 |  |
| Left precentral gyrus, mm | 2.6 ± 0.1 | 2.6 ± 0.1 | 0.834 |  |
| Right precentral gyrus, mm | 2.5 ± 0.1 | 2.5 ± 0.1 | 0.573 |  |
| Left precuneus, mm | 2.3 ± 0.1 | 2.4 ± 0.1 | 1.000 |  |
| Right precuneus, mm | 2.4 ± 0.1 | 2.4 ± 0.1 | 0.744 |  |
| Left rostral anterior cingulate cortex, mm | 2.4 ± 0.1 | 2.4 ± 0.2 | 0.733 |  |
| Right rostral anterior cingulate cortex, mm | 2.5 ± 0.2 | 2.5 ± 0.1 | 0.995 |  |
| Left rostral middle frontal gyrus, mm | 2.4 ± 0.1 | 2.4 ± 0.1 | 0.779 |  |
| Right rostral middle frontal gyrus, mm | 2.4 ± 0.1 | 2.4 ± 0.1 | 0.248 |  |
| Left superior frontal gyrus, mm | 2.8 ± 0.1 | 2.8 ± 0.1 | 0.744 |  |
| Right superior frontal gyrus, mm | 2.8 ± 0.1 | 2.8 ± 0.1 | 0.374 |  |
| Left superior parietal lobule, mm | 2.2 ± 0.1 | 2.2 ± 0.1 | 0.483 |  |
| Right superior parietal lobule, mm | 2.2 ± 0.1 | 2.2 ± 0.1 | 0.425 |  |
| Left superior temporal gyrus, mm | 2.4 ± 0.1 | 2.4 ± 0.2 | 0.222 |  |
| Right superior temporal gyrus, mm | 2.5 ± 0.2 | 2.6 ± 0.2 | 0.264 |  |
| Left supramarginal gyrus, mm | 2.4 ± 0.1 | 2.4 ± 0.1 | 0.818 |  |
| Right supramarginal gyrus, mm | 2.5 ± 0.1 | 2.5 ± 0.1 | 0.636 |  |
| Left frontal pole, mm | 2.4 ± 0.2 | 2.5 ± 0.2 | 0.435 |  |
| Right frontal pole, mm | 2.4 ± 0.1 | 2.4 ± 0.2 | 0.444 |  |
| Left temporal pole, mm | 3.1 ± 0.2 | 3.1 ± 0.2 | 0.343 |  |
| Right temporal pole, mm | 3.0 ± 0.3 | 3.0 ± 0.2 | 0.714 |  |
| Left transverse temporal gyrus, mm | 2.4 ± 0.1 | 2.4 ± 0.1 | 0.605 |  |
| Right transverse temporal gyrus, mm | 2.4 ± 0.1 | 2.5 ± 0.2 | 0.301 |  |
| Left insular cortex, mm | 2.7 ± 0.2 | 2.7 ± 0.1 | 0.249 |  |
| Right insular cortex, mm | 2.5 ± 0.1 | 2.8 ± 0.1 | **0.012** |  |

Data expressed as mean ± SD

Significant differences after the Benjamini-Hochberg correction are marked in bold
